# Prevalence of brucellosis in the human, livestock and wildlife interface areas of Serengeti National Park, Tanzania

**DOI:** 10.4102/ojvr.v83i1.1032

**Published:** 2016-05-24

**Authors:** Gabriel M. Shirima, John S. Kunda

**Affiliations:** 1 Department of Health and Biomedical Sciences, The Nelson Mandela African Institution of Science and Technology, Tanzania; 2 National Institute of Medical Research, Ministry of Health, Tanzania

## Abstract

Between 2005 and 2006, a cross-sectional survey was carried out in domestic ruminants in agropastoral communities of Serengeti district, Tanzania to determine the seroprevalence of brucellosis in domestic–wildlife interface villages. Both the Rose Bengal Plate Test (RBPT) and Competitive Enzyme Linked-immunosorbent Assay (c-ELISA) were used to analyse 82 human and 413 livestock sera from four randomly selected villages located along game reserve areas of Serengeti National Park. Although both cattle (288) and small ruminants (125) were screened, seropositivity was detected only in cattle. The overall seroprevalence based on c-ELISA as a confirmatory test was 5.6%. In cattle both age and sex were not statistically associated with brucellosis seropositivity (*P* = 0.63; 95% CI = 0.03, 0.8 and 0.33; 95% CI = 0.6, 3.7, respectively). Overall herd level seropositivity was 46.7% (*n* = 7), ranging from 25% to 66.7% (*n* = 4–10). Each village had at least one brucellosis seropositive herd. None of the 82 humans tested with both RBPT and c-ELISA were seropositive. Detecting *Brucella* infection in cattle in such areas warrants further investigation to establish the circulating strains for eventual appropriate control interventions in domestic animals.

## Introduction

Brucellosis is caused by a variety of *Brucella* species and is a disease of major socio-economic importance in domestic animals worldwide, especially in developing countries where disease control programmes are either non-existent or inadequate. The disease also occurs in wild animals, thus posing a danger of transmission between domestic and wild animals in wildlife–livestock interface areas (Assenga *et al*. 2015; Shirima 2005; Shirima *et al*. 2007). *Brucella* species that cause disease in terrestrial animals include *Brucella abortus*, *Brucella melitensis*, *Brucella suis*, *Brucella ovis* and *Brucella canis* (Corbel 1988). *B. abortus*, *B. melitensis*, *B. suis* and *B. canis* can all cause human brucellosis, with different degrees of severity. Serengeti is one of the districts housing national parks in Tanzania. Serengeti National Park occupies more than 50% of the area of Serengeti district. Livestock keepers in Serengeti district who entirely depend on livestock production and practise limited agriculture observed domestic animals commingling with wildlife throughout the year in search of pastures and water. This is also common in villages located at interface areas of Serengeti National Park (Fyumagwa 2012). The presence of zoonotic diseases in domestic animals around the Serengeti ecosystem could be a source of cross-transmission to wildlife because commingling between the two populations has been reported elsewhere (Assenga *et al*. 2015; Fyumagwa *et al.* 2009; Shirima *et al.* 2007). *Brucella* infection has been reported in wildebeests (*Connochaetes taurinus*), buffaloes (*Syncerus caffer*) (Fyumagwa *et al.* 2009), and cattle (Bugwesa *et al*. 2009) in the Serengeti ecosystem, but has not been reported from small ruminants and humans from the same interface areas. Assenga *et al*. (2015) and Fyumagwa *et al*. (2009) suggested further studies in livestock and human populations adjacent to wildlife areas and characterisation of the circulating pathogens for future interventions. Therefore, this seroprevalence study was conducted to elucidate the disease extent and distribution in livestock and humans in villages located in the wildlife–livestock interface area in Serengeti district so as to devise control and preventive strategies.

## Materials and methods

### Study location

The study was conducted in Serengeti district, Tanzania. The district has an area of 10 373 km^2^ with a human population of about 249 420 (NBS 2012). The Serengeti National Park occupies about 7000 km^2^ whereas the game reserve occupies 258 km^2^ of its area. The human population occupies only 659 km^2^, including farming and livestock practices.

### Study villages

Four villages, namely Bisarara, Ketembere, Masinki, and Nyichoka, bordering game reserves around Serengeti National Park were selected randomly for the study between November 2005 and March 2006. Four herds were selected randomly from each village for sampling with the exception of one village where three herds were selected. In these villages, cattle and small ruminants were kept together and thus one household was considered to constitute one herd.

### Sample size

The livestock sample size was estimated to provide 80% power of getting a seropositive animal with 95% confidence. Based on an estimate of 50% brucellosis prevalence and 0.05 error margin, the sample size was calculated as described by Martin, Meek and Willeberg (1987) to obtain the total number of domestic ruminants to be screened from the district:

$$n=\frac{z^{2}\times P(1-P)}{d^{2}}$$ [Eqn 1]

where: *n* = the required sample size; *P* = estimated prevalence = 0.5; *z* = level of confidence as 1.96 and *d* = desired precision level = 0.05.

### Blood sampling and processing

#### Livestock sampling

It was estimated to sample at least 100 animals from four herds in each village with 25–30 animals per herd. The animals were sampled randomly from herds with more than 30 animals whereas in herds with 1–30 animals, all were tested.

#### Blood sample and data collection from animals

Whole blood (10 mL) was collected in plain vacutainers from each animal. Individual bio-data such as sex, age, species, and abortion history in the previous year were collected. Blood samples were processed on the day of collection. Samples were centrifuged for 5 min using a mobile spin centrifuge (Vulcan Technologies, USA) at the Serengeti Veterinary Office. Serum was aliquoted into Eppendorf tubes (Eppendorf-Netheler-Hinz GmbH, Hamburg, Germany) in duplicate and kept at -20 °C. All serum samples were subjected to the Rose Bengal Plate Test (RBPT) as screening test at Sokoine University of Agriculture, Tanzania. RBPT-positive samples were confirmed by competitive enzyme-linked immunosorbent assay (c-ELISA). The kits and antigen used for screening and confirmation were kindly donated by the Animal and Plant Health Agency (APHA), Weybridge, UK (batch numbers SG269 and SG276). The agglutination test and c-ELISA were performed and interpreted as described by APHA protocol (Perret *et al*. 2001). The ELISA test results were measured by ELISA reader Multiscan RC Version 6.0 (Labsystems, Helsinki, Finland) at 450 nm. The plate results were considered invalid if any of the following applied:

- The binding ratio was less than 10.
- The optical density (OD) of the mean of the 6 negative ODs was less than 0.70. The optimal mean negative OD was 1.0.
- The OD of the mean of the 6 positive wells was greater than 0.10.
- The mean OD of the four conjugate control wells was less than 0.70 (Shirima 2005).

The cut-off value for c-ELISA positivity was based on the conjugate control where the cut-off was taken as 60% of the mean of the OD of the four conjugate control wells. Any test sample giving an OD equal to, or below this value, was considered positive. All results were expressed as a percentage of the conjugate control and referred to as percentage positive values (Shirima 2005).

#### Blood collection from humans

In each livestock-keeping household, family members were approached to identify volunteers for blood samples following discussions about the purpose of the project and the potential of the brucellosis problem in the district. Blood was aseptically collected from the brachial vein using a disposable 5 mL-syringe (Ha Young Corporation, Korea). The blood was immediately transferred into a plain vacutainer and serum separation and testing was processed as described in the previous section (Shirima 2005).

### Statistical analysis

The data collected were stored and analysed using version 7.1.1.14 of the Epi-Info software package (Centres for Disease Control and Prevention, Atlanta, GA, USA). Seroprevalence with 95% confidence interval was calculated for individual and herd level estimates. The relationship between dependent variables (herd and animal level) and outcome (serostatus) was explored using the Mantel–Haenzel test (Epi-Info software 2015).

## Ethical consideration

Blood collection from humans was carried out after obtaining ethical clearance from the Ministry of Health and Social Welfare, Tanzania (Ethical Clearance number NIMR/HQ/Vol. iv/B575). Sampling of livestock was done with verbal consent from the herd owners who were informed about the purpose of the project.

## Results

### Herd structure

Both cattle and small ruminants (goats and sheep) constituted a herd, as they stayed together. The herd size ranged from 9 to 150 domestic ruminants. The majority of herds (80%) had a herd size between 9 and 40 animals, with one herd having more than 100 animals. In the study area; 296 cattle, 75 goats and 42 sheep were screened for *Brucella* antibodies.

### Livestock seropositivity

The prevalence of brucellosis in domestic ruminants based on c-ELISA was 5.6% in livestock–wildlife interface areas of Serengeti district. Although cattle and small ruminants were kept together, seropositivity was only detected in cattle. There was no significant difference (*P* = 0.63) in seropositivity between female (3.2%) and male (3.8%) animals from this study. In addition, seropositivity was not statistically influenced by age between young animals (≤ 2.5 years, *n* = 159) and adults (> 2.5 years, *n* = 254) or sex (*P* = 0.33; *P* = 0.63). The overall *Brucella* seropositivity at herd level was 46.7%, although it ranged from 25% to 66.7%. Village level seropositivity ranged from 1.9% to 11% with highest level observed in Nyichoka village (11%), followed by Bisarara (8.6%), and the lowest seroprevalence was observed in Ketembere village (1.9%) (Table 1).

**TABLE 1:** Herd and individual level Brucella seropositivity in selected villages of Serengeti district.

Village	Herds screened	% of seropositive herds	95% CI	Animals screened	Seropositive animals	Percentage of individual seropositivity	95% CI
Bisarara	4	50	0.01, 0.99	70	6	8.6	0.02, 0.15
Ketembere	4	50	0.01, 0.99	155	3	1.9	0.00, 0.04
Masinki	3	67	0.13, 1.20	124	7	5.6	0.02, 0.09
Nyichoka	4	25	-0.17, 0.67	64	7	11	0.03, 0.19

**Total**	**15**	**46.7**	**0.21, 0.72**	**413**	**23**	**5.6**	**0.03, 0.08**

Note: Herd includes cattle and small ruminants as they are kept together.

Between the years 2005 and 2006, 20 cases of abortions were reported from the study herds with one seropositive herd at Masinki village experiencing seven cases of abortion. However, there was no statistically significant association between animal level c-ELISA seropositivity and abortion (*P* = 0.66, CI = 0.02, 7.2).

### Human *Brucella* seropositivity

In total, 82 humans (47 males and 35 females) volunteered for brucellosis screening. None tested positive with either RBPT or c-ELISA.

## Discussion

The serological survey conducted revealed that only cattle from the four villages surveyed were exposed to *Brucella* infection. The individual and herd seroprevalence of 5.6% and 46.6% noted from this study were comparable to other studies in agropastoral and pastoral communities in Tanzania (Assenga *et al*. 2015; Bugwesa *et al*. 2009; Shirima *et al.* 2007). The infection in cattle suggests exposure to the bacteria because vaccination using S19 is not practised in Tanzania, including Serengeti district. Infection in cattle residing close to wildlife areas could perpetuate transmission between cattle and wildlife animals and thus maintain the disease in the two populations.

*Brucella* seropositivity was not detected in sheep and goats in the study area despite the fact that all the animals were kept together. This could be attributed to the extent of environmental contamination and duration of the infection in the study herds. These findings were comparable to those of Assenga *et al*. (2015), where seropositivity was significantly low in small ruminants compared to cattle. However, several studies have indicated *Brucella* infection in both cattle and small ruminants that cohabited (Gamal *et al*. 2014; Shirima 2005). Although cattle and small ruminants from the same herd could be exposed to *Brucella* infection, no study has been conducted to elucidate the circulating *Brucella* species.

Encountering *Brucella* seropositivity in all villages could indicate the spatial distribution of the disease and the potential to perpetuate the disease within and between villages, depending on how densities and contact rates among animals increases. Similar observations were reported by Jiwa *et al*. (1996), Kadohira *et al*. (1997), and Shirima (2005), who suggested that commingling of herds at grazing and watering points were risk factors for disease transmission.

## Conclusion

In conclusion, anti-*Brucella* antibodies were present in cattle in the Serengeti ecosystem, suggesting that the infection might circulate in the ecosystem. Therefore, further studies are required to establish spatial and temporal distribution and characterise *Brucella* species for eventual control interventions in livestock.
